# The first species of the pseudoscorpion genus *Lechytia* Balzan, 1892 (Pseudoscorpiones, Chthoniidae) from New Zealand

**DOI:** 10.3897/zookeys.1000.56313

**Published:** 2020-12-03

**Authors:** Jana Christophoryová, Katarína Krajčovičová

**Affiliations:** 1 Department of Zoology, Faculty of Natural Sciences, Comenius University, Mlynská dolina, Ilkovičova 6, SK–842 15 Bratislava, Slovakia Comenius University Bratislava Slovakia

**Keywords:** description, *Lechytia novaezealandiae*, Pacific Ocean, taxonomy

## Abstract

The subfamily Lechytiinae is reported from New Zealand for the first time. A new species, *Lechytia novaezealandiae***sp. nov.**, is described and illustrated from Lake Waikare in Waikato District, North Island. In addition, a key to species in the genus *Lechytia* from Asia, Australia, and New Zealand is included.

## Introduction

Lechytiines were first recognised as a tribe of the Chthoniidae by Chamberlin (1929). Muchmore (1975) suggested that this species group may actually deserve subfamily or even family rank. Harvey (1992) removed Lechytiini from the Chthoniidae and elevated it to the family level and regarded the lack of an elliptical areole on the rallum and the short inter-maxillary jugum as diagnostic. However, one of the results of the most recent large phylogenomic analysis was the revised rank for Lechytiidae, which is regarded as a subfamily of Chthoniidae (Benavides et al. 2019) and includes 25 species in a single genus (Harvey 2013; Zhang and Zhang 2014). One of them, *Lechytia tertiaria* Schawaller, 1980, is a fossil Oligocene species from the Dominican Republic (Schawaller 1980).

Lechytiines occur in most regions of the world but particularly in tropical and subtropical zones. Most of them have restricted distributions, being only known from a few locations (Harvey 2013). Twelve species are known from the Americas (including the fossil one), six from Africa, one from each of Turkey and Australia, and six from Asia, including the Pacific region (Harvey 2013; Zhang and Zhang 2014). However, they are small, easily overlooked, and seldom collected, and therefore, the actual distribution of *Lechytia* is still unknown (Muchmore 1975). This is evidenced by the fact that the genus was not long ago recorded in Australia or China, where both most recently published records (Harvey 2006; Zhang and Zhang 2014) represented new species.

Lechytiines are often corticolous, living under or between the bark of trees and in tree hollows (e.g. Beier 1965; Muchmore 1975; Harvey 2006; Zhang and Zhang 2014), but they have also been found in soil, litter, or moss (e.g. Beier 1955a; Muchmore 1975; Mahnert 1978; Zhang and Zhang 2014), in caves on bat guano (e.g. Beier 1970; Muchmore 1973) and in termite nests (Beier 1959). *Lechytia sakagamii* Morikawa, 1952 was collected from an albatross nest (Muchmore 2000). This species has been reported from a number of islands in the Pacific Ocean, and Muchmore (2000) presumed that it is likely to be phoretic on sea birds and that humans have also transported it.

We have received two *Lechytia* specimens and discovered that they represent the first record of the subfamily Lechytiinae in New Zealand. The new discovery led us to provide a description of the new species, here called *Lechytia novaezealandiae*.

## Material and methods

Both specimens of *Lechytia novaezealandiae* sp. nov. examined for this study had been preserved in 75% ethanol. They were studied as temporary slide mounts, prepared by immersing of the specimens in lactic acid for clearing. After the study, they were rinsed in water and returned to 75% ethanol, with the dissected portions being placed in microvials.

Morphological and morphometric analyses were performed using a Leica DM1000 compound microscope with an ICC50 Camera Module (LAS EZ application, 1.8.0). Measurements were taken from digital images (photographed using a Leica DM2500 compound microscope with a Canon EOS 70D camera) using the AxioVision 40LE application. Reference points for measurements follow Chamberlin (1931). Drawings were generated using a Leica DM1000 drawing tube. Digital photograph of the new species was taken using a Canon EOS 5D camera attached to a Zeiss Axio Zoom V16 stereomicroscope. Image stacks were produced manually, combined using the Zerene Stacker software and edited with Adobe Photoshop CC.

The terminology follows Harvey (1992), except for the use of the terms rallum (Judson 2007) and duplex trichobothria (Judson 2018).

The types of the new species are deposited in the zoological collection of the Museum of New Zealand Te Papa Tongarewa, New Zealand.

## Taxonomy

**Family Chthoniidae Daday, 1889**

**Subfamily Lechytiinae Chamberlin, 1929**

### Genus Lechytia

Taxon classificationAnimaliaPseudoscorpionesChthoniidae

Balzan, 1892

95F828C8-0484-5273-B17D-B7C1ED8E1CF6

#### Type species.

*Roncus chthoniiformis* Balzan, 1887, by original designation.

#### Diagnosis.

For the members of Lechytiinae, the most peculiar diagnostic feature is the arrangement of the trichobothria *eb* and *esb* on the chelal hand dorsum (in all other chthoniids, these trichobothria are situated at the base of the fixed chelal finger) (Harvey 2006).

### Lechytia novaezealandiae sp. nov.

Taxon classificationAnimaliaPseudoscorpionesChthoniidae

628302FA-3438-5B20-A2AB-1A74FE50C965

http://zoobank.org/84886B8E-DE63-4ABA-963B-3CF59C2A87F5

Figures 1 , 2 , 3

#### Material examined.

***Holotype***: New Zealand • ♂; North Island, Waikato District, near Lake Waikare [-37.456, 175.189]; 5 m a.s.l.; 25 Jul. 1980; Galina Fedorovna Kurcheva leg.; moss; AF.000964. ***Paratype***: • ♀; same data as holotype; AF.000965.

**Figure 1.**
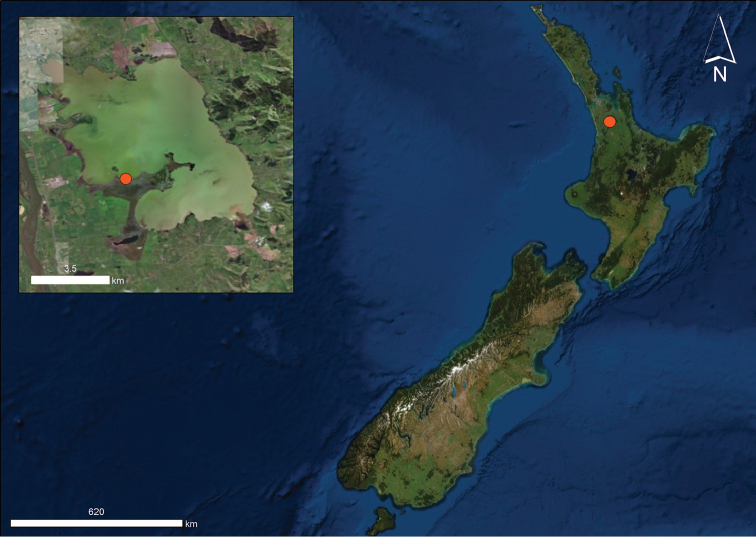
Distribution of *Lechytia novaezealandiae* sp. nov. in New Zealand (orange circle).

#### Diagnosis.

The new species belongs to the “*arborea*” group and is characterised by the following combination of characteristics: trichobothria *b* and *sb* situated less than 1 areolar diameter apart; palpal chela 3.17–3.30× and palpal hand 1.58–1.60× longer than broad; palpal femur 0.21–0.24 mm, palpal hand 0.16–0.19 mm and chelal moveable finger 0.19–0.22 mm long.

#### Description.

**Figure 2.**
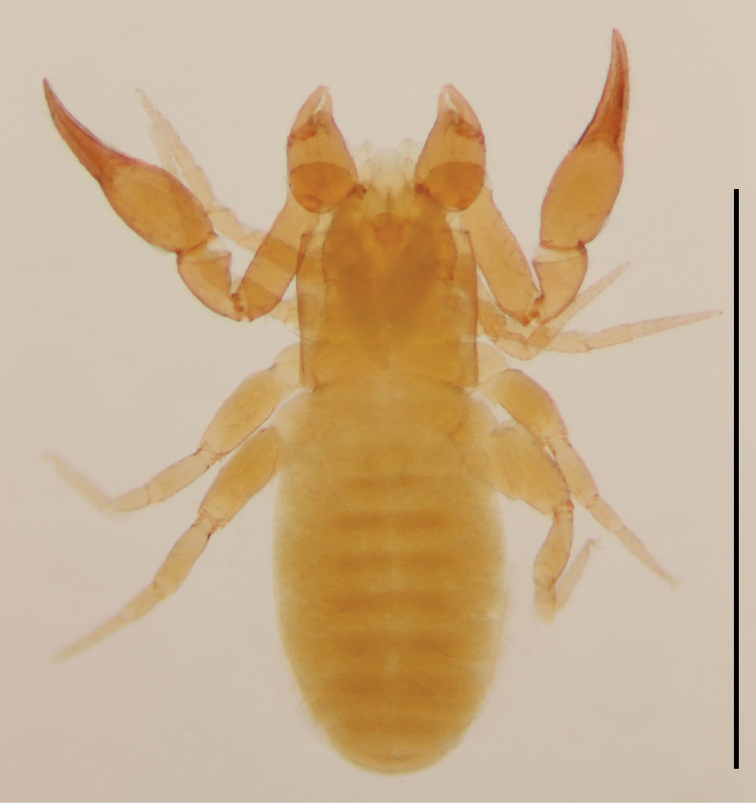
*Lechytia novaezealandiae* sp. nov., paratype female, dorsal. Scale bar: 1 mm.

**Figure 3.**
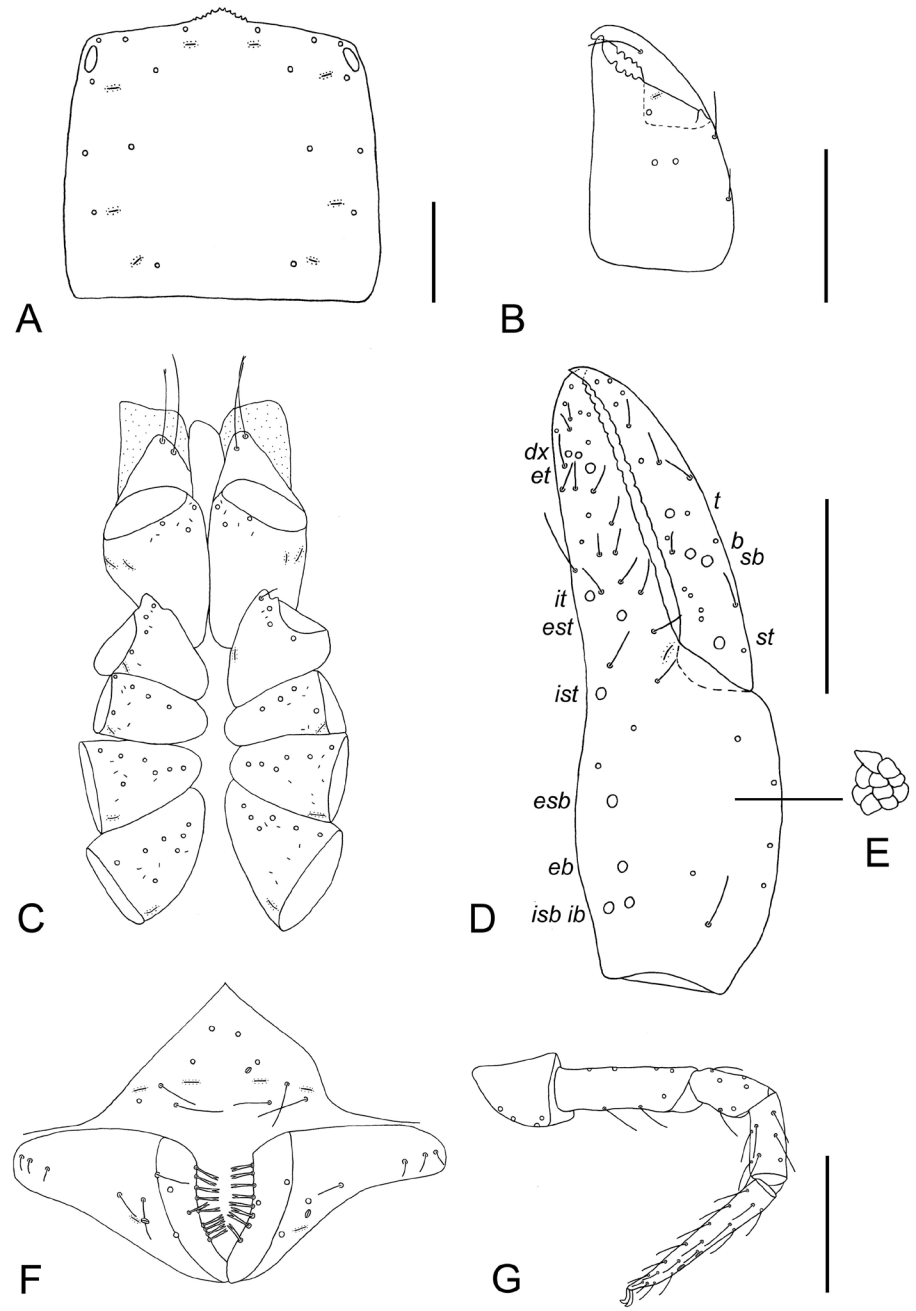
*Lechytia novaezealandiae* sp. nov., adults, dorsal **A** carapace (female) **B** right chelicera (male) **C** coxae (male) **D** right chela, showing trichobothrial pattern (male) **E** detail of structure on palpal hand **F** chaetotaxy of genital area (sternites II–III) (male) **G** Right leg I (female). Abbreviations: moveable chelal finger: *t*–terminal, *b*–basal, *sb*–subbasal, *st*–subterminal; fixed chelal finger: *dx*–duplex trichobothria, *et*–exterior terminal, *it*–interior terminal, *est*–exterior subterminal, *ist*–interior subterminal, *esb*–exterior subbasal, *eb*–exterior basal, *isb*–interior subbasal, *ib*–interior basal. Scale bars: 0.1 mm.

**Adults** (Figs 2, 3). ***Carapace*** (Fig. 3A): 1.08× (♂), 0.94× (♀) longer than broad; with two small corneate eyes; anterior margin denticulate, more markedly in female; in female with 18 setae arranged 6: 4: 4: 2: 2, in male with 17 setae arranged 6: 4: 3: 2: 2; without furrows; with four pairs of lyrifissures, first pair situated antero-medially, the second pair situated interno-lateral to the eyes, the third pair situated slightly interior to the sole pair of setae of the intermediate row, and the fourth pair situated exterior to the sole pair of setae of the posterior row (Fig. 3A). ***Coxae*** (Fig. 3C): manducatory process with two distal setae, about equal in length, the distal setae terminally bifurcate (Fig. 3C); coxal spines and intercoxal tubercle absent; chaetotaxy of coxae (Fig. 3C): palpal coxae 3 (♂, ♀); pedal coxae I 4 (♂), 3–4 (♀); coxae II 4–5 (♂), 5 (♀); coxae III 6–7 (♂, ♀); coxae IV 6–7 (♂), 7 (♀); coxa I with small, triangular apical projection with single seta situated at base, other setae on coxa I situated near trochanteral foramen (Fig. 3C). For lyrifissures, see Fig. 3C. ***Chelicera*** (Fig. 3B): 1.50× (♂), 1.67× (♀) longer than broad; five acuminate setae and one lyrifissure on hand; moveable finger with one medial seta; both fixed and moveable finger with four (♂) or five (♀) teeth, the distal-most tooth on both fingers largest; galea of ♂ absent, that of ♀ a short rounded nubbin; serrula exterior with 15 blades; rallum consisting of seven blades, subdistal blade strongly recumbent, others in straight row. ***Pedipalp*** (Fig. 3D): all setae acuminate; trochanter 1.57× (♂), 1.71× (♀); femur 3.00× (♂, ♀); patella 1.71× (♂), 1.67× (♀); chela 3.30× (♂), 3.17× (♀); hand 1.60× (♂), 1.58× (♀) longer than broad. Fixed chelal finger and hand with eight trichobothria, *ib*, *isb*, *eb* and *esb* on dorsum of hand, *ib* and *isb* basally, *esb* medially, *eb* closer to *ib* and *isb* than to *esb*; *ist*, *est* and *it* situated basally on fixed finger, *et* and *dx* distally; moveable chelal finger with four trichobothria, *b* closer to *sb* than to *t*; *b* and *sb* less than one areolar diameter apart; sensilla absent (Fig. 3D). Venom apparatus absent. Fixed and moveable finger with approximately 9–12 distal small teeth followed by remaining obsolete teeth, fused into a lamina; accessory teeth absent (Fig. 3D). ***Opisthosoma***: tergites and sternites undivided; setae acuminate. Tergal chaetotaxy I–X: (♂, ♀) 6: 6: 6: 6: 6: 6: 6: 6: 6: 4: 1T2T1. Tergal lyrifissures I–X: (♂) 5: 4: 4: 6: 4: 4: 4: 4: 6: 2; (♀) 2: 2: 2: 4: 4: 2: 2: 2: 2: 4. Sternal chaetotaxy II–X: (♂) 10: 15: 12: 9: 8: 6: 6: 6: 6 (Fig. 3F); (♀) 8: 12: 12: 12: 9: 8: 8: 6: 6. All 19 setae bordering male sternite III bifurcate. Sternal lyrifissures II–X: (♂) 4: 2: 2: 2: 2: 2: 2: 1: 0; (♀) 2: 2: 2: 2: 2: 2: 2: 2: 0. Sternal pores II–X: (♂) 1: 2: 3: 5: 2: 2: 2: 2: 8; (♀) 2: 4: 6: 4: 3: 4: 4: 4: 8. Genitalia not studied in detail; those of female weakly sclerotised with U-shaped frame. ***Leg I***: trochanter 1.40× (♂, ♀); femur 3.67× (♂), 3.25× (♀); patella 1.50× (♂), 1.75× (♀); tibia 2.00× (♂), 2.33× (♀); tarsus 5.50× (♂), 4.67× (♀) deeper than broad. ***Leg IV***: trochanter 1.17× (♂), 1.14× (♀); femoropatella 1.91× (♂), 1.77× (♀); tibia 2.60× (♂), 3.00× (♀); metatarsus 2.00× (♂, ♀); tarsus 5.50× (♂), 4.33× (♀) deeper than broad. Legs robust, heterotarsate; tarsi with two elongate gland openings along the dorsal surface, each with crenulated margins (Fig. 3G); arolium slightly shorter than claws, claws simple.

#### Dimensions

(length/width or, in the case of the legs, length/depth) in mm. Body length 0.78 (♂), 0.97 (♀). Pedipalp: trochanter 0.11/0.07 (♂), 0.12/0.07 (♀); femur 0.21/0.07 (♂), 0.24/0.08 (♀); patella 0.12/0.07 (♂), 0.15/0.09 (♀); chela 0.33/0.10 (♂), 0.38/0.12 (♀); hand 0.16/0.10 (♂), 0.19/0.12 (♀); moveable finger 0.19 (♂), 0.22 (♀). Chelicera 0.15/0.10 (♂), 0.20/0.12 (♀); moveable finger 0.09 (♂), 0.10 (♀). Carapace 0.28/0.26 (♂), 0.30/0.32 (♀). Leg I: trochanter 0.07/0.05 (♂, ♀); femur 0.11/0.03 (♂), 0.13/0.04 (♀); patella 0.06/0.04 (♂), 0.07/0.04 (♀); tibia 0.06/0.03 (♂), 0.07/0.03 (♀); tarsus 0.11/0.02 (♂), 0.14/0.03 (♀). Leg IV: trochanter 0.07/0.06 (♂), 0.08/0.07 (♀); femoropatella 0.21/0.11 (♂), 0.23/0.13 (♀); tibia 0.13/0.05 (♂), 0.15/0.05 (♀); metatarsus 0.08/0.04 (♂, ♀); tarsus 0.11/0.02 (♂), 0.13/0.03 (♀).

#### Etymology.

The specific epithet refers to the island country of New Zealand, on which this species occurs.

#### Distribution and habitat.

*Lechytia novaezealandiae* sp. nov. Is at present known only from the type locality near Lake Waikare, Waikato District, North Island, New Zealand at an altitude of 5 m. The specimens were collected in moss in July.

## Comparisons

*Lechytia* species have rarely been studied in recent years, and little is known about the relationships between the named species (Zhang and Zhang 2014). Only few characteristics are available for most of them (Muchmore 2000). Two species-groups can be recognised in this genus (Muchmore 1975, 2000). The “*arborea*” species-group is characterised as follows: bifurcate distal seta on palpal coxa, strongly reduced chelal teeth, tergite XI with chaetotaxy 1T2T1, and male galea is reduced. The “*hoffi*” species-group is diagnosed as follows: simple distal seta on palpal coxa, well-developed chelal teeth, tergite XI with chaetotaxy T2T, and male galea nearly as well developed as in female (Muchmore 1975, 2000).

The “*hoffi*” group is presently known to include only two species – *Lechytia hoffi* Muchmore, 1975 from the United States and *L. yulongensis* Zhang & Zhang, 2014 from China (Muchmore 1975; Zhang and Zhang 2014). The “*arborea*” group includes the three American species *L. arborea*﻿ Muchmore, 1975, *L. sini*﻿ Muchmore, 1975, *L. chthoniiformis* (Balzan, 1887), one Asian species *L. sakagamii*﻿ Morikawa, 1952, and one Australian species *L. libita*﻿ Harvey, 2006 (Muchmore 1975, 2000; Mahnert 2001; Harvey 2006). The remaining species of the genus have not yet been placed into the two known species-groups.

*Lechytia novaezealandiae* sp. nov. also belongs to the “*arborea*” group and differs from all above-mentioned species from the “*arborea*” group by its smaller palpal dimensions (e.g. *L. arborea*﻿ femur 0.31–0.32, chela 0.50–0.52, moveable finger 0.27–0.28 mm; *L. sini*﻿ femur 0.25–0.30, chela 0.38–0.47, moveable finger 0.23–0.27 mm; *L. chthoniiformis*﻿ femur 0.30–0.32, chela 0.46, moveable finger 0.25–0.27 mm; *L. sakagamii* femur 0.27–0.30, chela 0.41–0.45, moveable finger 0.24–0.26 mm; *L. libita*﻿ femur 0.27–0.32, chela 0.40–0.46, moveable finger 0.24–0.28 mm; but *L. novaezealandiae* sp. nov. femur 0.21–0.24, chela 0.33–0.38, moveable finger 0.19–0.22 mm) (Beier 1932; Muchmore 1975, 2000; Mahnert 2001; Harvey 2006).

The new species differs from *L. indica*﻿ Murthy & Ananthakrishnan, 1977, *L. madrasica* Sivaraman, 1980 (both from India), and from *L. cavicola*﻿ Muchmore, 1973 (Mexico) by the presence of eyes on the carapace and smaller palpal femur and chela (Muchmore 1973; Murthy and Ananthakrishnan 1977; Sivaraman 1980).

From known African species, *L. novaezealandiae*﻿ sp. nov. differs by smaller palpal hand and finger, as well as by the position of trichobothria *sb* and *b* on moveable chelal finger (in *L. leleupi*﻿ Beier, 1959, *L. dentata*﻿ Mahnert, 1978, and *L. natalensis* (Tullgren, 1907) trichobothria *sb* and *b* are situated close together; in *L. serrulata*﻿ Beier, 1955 and *L. maxima*﻿ Beier, 1955, trichobothria *sb* and *b* are situated more than one areolar diameter apart) (Beier 1932, 1955a, 1955b, 1959; Mahnert 1978). The position of trichobothria *sb* and *b* is similar in *L. novaezealandiae*﻿ sp. nov. and *L. garambica* Beier, 1972, but they differ in palpal measurements (e.g. *L. garambica* femur 0.26–0.27, hand 0.20 mm or chela ratio 3.7–4.4× longer than broad, but in *L. novaezealandiae* sp. nov. femur 0.21–0.24, hand 0.16–0.19 mm or chela ratio 3.17–3.30× longer than broad) (Beier 1972).

The situation is similar for other known species from the Americas, Asia, and Turkey; *L. novaezealandiae* sp. nov. differs by smaller palpal segments (*L. delamarei* Vitali-di Castri, 1984 femur 0.32, finger 0.28 mm; *L. chilensis*﻿ Beier, 1964 hand 0.24, finger 0.33 mm; *L. trinitatis* Beier, 1970 femur 0.30, hand 0.23–0.24, finger 0.25–0.26 mm; *L. martiniquensis*﻿ Vitali-di Castri, 1984 femur 0.32, finger 0.29 mm; L. kuscheli Beier, 1957 hand 0.25–0.29, finger 0.33–0.39 mm; *L. himalayana*﻿ Beier, 1974 femur 0.50, hand 0.27, finger 0.34 mm; *L. asiatica* Redikorzev, 1938 femur 0.30, hand 0.20 mm; *L. anatolica*﻿ Beier, 1965 hand 0.24, finger 0.28 mm) (Redikorzev 1938; Beier 1957, 1964, 1965, 1970, 1974; Vitali-di Castri 1984). Additionally, in *L. chilensis*﻿, *L. kuscheli*, *L. himalayana*﻿, trichobothria *sb* and *b* are situated more than 1 areolar diameter apart (Beier 1957, 1964), while in *L. asiatica*, they are contiguous (Harvey 2006).

### Identification key to the species of *Lechytia* from Asia, Australia, and New Zealand

**Table:** 

1	Eyes or eyes spots absent	**2**
–	Eyes or eyes spots present	**3**
2	Pedipalps slender; palpal femur 3.05–3.10 times longer than broad; palpal chela 4.20–4.30 times longer than broad	***L. madrasica***
–	Pedipalps robust; palpal femur 2.20–2.30 times longer than broad; palpal chela 3.80–3.90 times longer than broad	***L. indica***
3	Trichobothria *b* and *sb* on moveable chelal finger situated less than 1 areolar diameter or even less apart	**4**
–	Trichobothria *b* and *sb* on moveable chelal finger situated 1 or more than 1 areolar diameter apart	**6**
4	Palpal femur shorter, 0.21–0.24 mm long	***L. novaezealandiae* sp. nov.**
–	Palpal femur longer, 0.27–0.30 mm long	**5**
5	Trichobothria *b* and *sb* on moveable chelal finger situated about half an areolar diameter apart; moveable chelal finger 0.24–0.26 mm long	***L. sakagamii***
–	Trichobothria *b* and *sb* on moveable chelal finger contiguous; moveable chelal finger 0.22 mm long	***L. asiatica***
6	Trichobothria *b* and *sb* on moveable chelal finger situated 2 areolas diameter apart	***L. himalayana***
–	Trichobothria *b* and *sb* on moveable chelal finger situated 1 areolar diameter apart	**7**
7	Distal seta on palpal coxa bifurcate; chelal teeth strongly reduced; tergite XI with chaetotaxy 1T2T1; male galea reduced (representative of “*arborea*” species-group)	***L. libita***
–	Distal seta on palpal coxa simple; chelal teeth well-developed; tergite XI with chaetotaxy T2T; male galea nearly as well developed as in female (representative of “*hoffi*” species-group)	***L. yulongensis***

## Supplementary Material

XML Treatment for Lechytia

XML Treatment for Lechytia novaezealandiae
